# Alpha‐lipoic acid alleviates NAFLD and triglyceride accumulation in liver via modulating hepatic NLRP3 inflammasome activation pathway in type 2 diabetic rats

**DOI:** 10.1002/fsn3.2235

**Published:** 2021-03-13

**Authors:** Chih‐Yuan Ko, Yangming Martin Lo, Jian‐Hua Xu, Wen‐Chang Chang, Da‐Wei Huang, James Swi‐Bea Wu, Cho‐Hua Yang, Wen‐Chung Huang, Szu‐Chuan Shen

**Affiliations:** ^1^ Department of Respiratory and Critical Care Medicine The Second Affiliated Hospital of Fujian Medical University Quanzhou China; ^2^ Department of Clinical Nutrition The Second Affiliated Hospital of Fujian Medical University Quanzhou China; ^3^ School of Public Health Fujian Medical University Fuzhou China; ^4^ Respiratory Medicine Center of Fujian Province Quanzhou China; ^5^ Institute for Advanced Study Shenzhen University Shenzhen China; ^6^ Department of Tumor Surgery The Second Affiliated Hospital of Fujian Medical University Quanzhou China; ^7^ Department of Food Science National Chiayi University Chiayi City Taiwan; ^8^ Department of Biotechnology and Food Technology Southern Taiwan University of Science and Technology Tainan City Taiwan; ^9^ Graduate Institute of Food Science and Technology National Taiwan University Taipei Taiwan; ^10^ Graduate Program of Nutrition Science National Taiwan Normal University Taipei Taiwan; ^11^ Graduate Institute of Health Industry Technology Chang Gung University of Science and Technology Taoyuan Taiwan

**Keywords:** alpha‐lipoic acid, NLRP3 inflammasome, nonalcoholic fatty liver disease, type 2 diabetes

## Abstract

The occurrence of nonalcoholic fatty liver disease (NAFLD) is associated with type 2 diabetes mellitus (T2DM). The activation of nucleotide‐binding domain and leucine‐rich‐containing family, pyrin domain‐containing 3 (NLRP3) inflammasome in the liver may lead to hepatic fat accumulation. Alpha‐lipoic acid (ALA) has been reported to improve IR in a T2DM rodent model. We investigated the effects of ALA on NLRP3 inflammasome activation and fat accumulation in the liver of a high‐fat diet (HFD) plus streptozotocin (STZ)‐induced T2DM rats. The HFD/STZ‐induced T2DM rats were orally administered ALA (50, 100, or 200 mg/kg BW) once a day for 13 weeks. The results showed that the liver triglyceride contents of T2DM rats were 11.35 ± 1.84%, whereas the administration of 50, 100, and 200 mg/kg BW ALA significantly reduced the liver triglyceride contents of T2DM rats to 4.14 ± 0.59%, 4.02 ± 0.41%, and 3.01 ± 1.07%, respectively. Moreover, 200 mg/kg BW ALA significantly decreased the hepatic levels of NLRP3 inflammasome activation‐related proteins NLRP3, caspase‐1, and interleukin‐1β expression by 40.0%, 60.1%, and 24.5%, respectively, in T2DM rats. Furthermore, the expression levels of hepatic fat synthesis‐related proteins decreased, namely a 45.4% decrease in sterol regulatory element‐binding protein‐1c, whereas the expression of hepatic lipid oxidation‐related proteins increased, including a 27.5% increase in carnitine palmitoyltransferase, in T2DM rats after 200 mg/kg BW ALA treatment. We concluded that ALA treatment may suppress hepatic NLRP3 inflammasome activation, consequently alleviating NAFLD and excess hepatic lipid accumulation in HFD/STZ‐induced T2DM rats.

## Graphical abstract

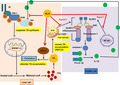

## 1 INTRODUCTION

Diabetes mellitus (DM) is a worldwide, chronic, noncommunicable disease with a complex etiology. To date, the popularity of Westernized diet, reduced physical activity, and aging populations appears to lead to the increasing prevalence of DM. Up to 2017, the number of people with DM has increased to 422 million and is expected to grow to 642 million over the next 25 years (Ogurtsova et al., 2017). Insulin resistance (IR) is the primary trait of type 2 DM (T2DM), which is known to result in the inability of peripheral cells to effectively uptake glucose, hence leading to hyperglycemia (Samuel & Shulman, 2012).

A chronic liver disease characterized as universal liver fat deposition without alcohol intake (Chalasani et al., 2012), nonalcoholic fatty liver disease (NAFLD) is one of the most common comorbidities of T2DM. Epidemiological research has reported that the prevalence of NAFLD in Western countries ranges from 20% to 30% versus 5%–18% in Asia (Benedict & Zhang, 2017). Clinically, 70%–90% of NAFLD patients are also diagnosed with T2DM or IR (Kotronen & Yki‐Järvinen, 2008). The progression of NAFLD has been associated with hepatic inflammation due to hyperglycemia. Lipid accumulation in the liver leads to hepatic fatty lesions, promotes the expression of inflammatory factors and IR, and eventually results in irreversible damage to the liver tissues (Shoelson et al., 2006).

Hepatic Kupffer cells are specialized macrophages that eliminate viruses and bacteria and rapidly trigger liver inflammation (Olteanu et al., 2014). The nucleotide‐binding domain, leucine‐rich‐containing family, pyrin domain‐containing 3 (NLRP3) is the most fully characterized inflammasome among others such as the adaptor protein apoptosis‐associated speck‐like protein (ASC) and the proinflammatory caspase, caspase‐1. The activation of the NLRP3 inflammasome stimulates Kupffer cells to secrete inflammatory cytokines, including interleukin (IL)‐1β, in the liver (Odegaard & Chawla, 2008). In addition, the activation of NLRP3 has also been associated with chronic diseases such as T2DM and NAFLD (Wang et al., 2018; Zhu et al., 2018).

Alpha‐lipoic acid (ALA), a vitamin‐like compound, is a thioctic acid and a sulfur‐containing organic compound that is enriched in the livers, kidneys, and hearts of animals. It can also be found in whole grains, spinach, cauliflower, and yeast (Singh & Jialal, 2008). ALA has been demonstrated to improve hyperglycemia, metabolic disorders, and liver inflammation (Abdelhalim et al., 2018; Castro et al., 2019). However, investigations of the effects of ALA on hepatic NLRP3 inflammasome activation and NAFLD have been limited. The present study aimed to elucidate the effects of ALA on hepatic NLRP3 inflammasome activation, fat accumulation, and NAFLD using a high‐fat diet (HFD) plus streptozotocin (STZ)‐induced T2DM model rats.

## 2 MATERIALS AND METHODS

### 2.1 Animal experimental procedures

Eight‐week‐old male Wistar rats, from the National Laboratory Animal Center (Taipei, Taiwan), were reared in the animal room of Taiwan Normal University. The room conditions and treatment procedures were in accordance with the National Institutes of Health (NIH) Guide for the Care and Use of Laboratory Animals, and all protocols were approved by the Institutional Animal Care and Use Committee of National Taiwan Normal University, Taipei, Taiwan (Approval Number 106,042). The rats were housed in a temperature‐controlled (22 ± 1°C) and humidity‐controlled (50 ± 20%) room, under a 12‐hr light/dark cycle (lights on from 08:00 to 20:00), with free access to food and water.

The induction of the diabetic rat model was performed as described by our previous study (Ko et al., 2019). After adapting to the environment for 1 week, the rats were fed an HFD (60% calories from fat) or a normal diet for 4 weeks. HFD‐fed rats were then intraperitoneally injected with STZ (Sigma, St. Louis, MO, USA) [30 mg/kg body weight (BW)], to induce diabetes, and then fed HFD for 9 more weeks. Thirty‐six rats were randomly divided into six groups, 6 rats in each: Normal group contained rats fed with normal diet; the DM group contained diabetic rats fed with HFD alone as the negative control; the DM + Pio group contained diabetic rats fed with HFD and orally administered pioglitazone (Pio, 30 mg/kg BW) daily for 13 weeks, as the positive control; and the DM + ALA50, DM + ALA100, and DM + ALA200 groups contained diabetic rats daily fed with HFD and orally administered 50, 100, or 200 mg/kg BW ALA (Sigma, St. Louis, MO, USA), respectively, for 13 weeks. All rats were sacrificed at the end of the experiment.

### 2.2 Blood sample collection

After the rats were sacrificed, blood was collected and centrifuged at 12,000 g for 8 min to obtain the serum samples, which were stored at 80°C before use.

### 2.3 Measurement of serum glucose and insulin

Glucose and insulin levels were measured by enzyme‐linked immunosorbent assay (ELISA) kits (Mercodia AB and Uppsala, Sweden), respectively. Biochemical analyses were conducted according to the supplier's protocols.

### 2.4 Measurement of plasma triglyceride (TG) and free fatty acid (FFA)

Levels of TG and FFA were measured by the commercial rat ELISA TG and FFA Kits (Crumlin, Co., Antrim, UK). The operation of biochemical assay followed protocols by Randox Laboratories.

### 2.5 Analysis of TG in the liver

The liver tissue was separated and removed immediately after sacrifice and stored at − 80°C until use. A 0.05 g liver tissue and 0.7 g homogenous beads were mixed with 0.5 ml standard diluent asset reagent (1×) and then added with a protease inhibitor to homogenize the tissue for three times (each 10 s per time). After 1 min in the ice bath, the homogenate was centrifuged at 10,000 g, 4°C for 30 min, then standing on ice bath again for 30 mins. The supernatant was taken and analyzed with commercial TG Assay Kit (Crumlin, Co., Antrim, UK) according to supplier's protocol.

### 2.6 Liver tissue protein preparation and measurement

To prepare liver protein samples, 0.1 g liver tissue was mixed with 0.5 ml lysis buffer and ground three times (10 s each time). After 1‐hr incubation in an ice bath, the homogenized tissue solutions were centrifuged at 20,000 g under 4°C for 40 min to obtain liver tissue protein supernatants. The protein concentration was measured at 595 nm using the Bradford method with a Bio‐Rad Protein Assay Kit (Hercules, CA, USA).

### 2.7 Western blot

Aliquots of liver tissue protein samples, each containing 40 µg of protein, were evaluated for the expression of NLRP3, apoptosis‐associated speck‐like protein containing CARD (ASC), caspase‐1, IL‐1β, nuclear factor kappa B (NF‐κB), phosphoinositide 3‐kinase (PI3K), protein kinase B (Akt/PKB), pAkt, sterol regulatory element‐binding protein‐1 (SREBP1), and carnitine palmitoyltransferase 1 (CPT‐1). The samples were subjected to 10% sodium dodecyl sulfate–polyacrylamide gel electrophoresis. The separated proteins were electrotransferred to a polyvinylidene difluoride membrane. The membrane was incubated with blocking buffer [phosphate‐buffered saline (PBS) containing 0.05% Tween‐20 (PBST) and 5% (wt/vol) nonfat dry milk] for 1 hr, incubated overnight at 4°C with PBST, and probed with anti‐NLRP3, anti‐ASC, anti‐caspase‐1, anti‐IL‐1β, anti‐NF‐κB, anti‐PI3K, anti‐Akt, anti‐pAkt, anti‐SREBP1, and anti‐CPT‐1 antibodies (1:1,000; Signalway Antibody, College Park, MD, USA). A 1:5,000 dilution of mouse monoclonal α‐tubulin antibody (Signalway Antibody; College Park, MD, USA) was used to ensure that a constant amount of protein was loaded into each lane of the gel. The membrane was washed 3 times (5 min each) in PBST, shaken in a solution of horseradish peroxidase‐conjugated anti‐mouse IgG or anti‐rabbit IgG (Genetex, Irvine, CA, USA) secondary, washed 3 times (5 min each) in PBST, and incubated in enhanced chemiluminescence reagent (Millipore, Darmstadt, Germany). Autoradiography was scanned and analyzed using a UVP Biospectrum Image System (Level, Cambridge, UK).

### 2.8 Statistical analyses

Values are presented as the mean ± standard deviation using SPSS version 19.0 (SPSS Inc., Chicago, IL, USA) by one‐way ANOVA, followed by Duncan's multiple comparisons tests. *p* < .05 was considered statistically significant.

## 3 RESULTS

### 3.1 Effects of ALA on fasting serum glucose and serum insulin, fasting plasma TG, and FFA levels in T2DM rats

Table 1 shows the selected fasting serum and plasma biochemistry analysis in HFD‐ and STZ‐induced T2DM rats treated with ALA for 13 weeks. The fasting serum glucose level of the DM group (274.1 ± 20.1 mg/dl) was significantly increased compared with that in the normal group (76.4 ± 6.4 mg/dl). Treatment with ALA (50 mg/kg BW, 100 mg/kg BW, or 200 mg/kg BW) decreased respective fasting serum glucose slightly relative to the level of the DM group without significant difference. A high level of fasting serum insulin (1.72 ± 0.27 µg/L) was also observed in the DM group. Serum insulin levels were significantly decreased in the DM + ALA50, DM + ALA100, and DM + ALA200 groups, respectively, compared with that in the DM group (Table 1). In addition, plasma TG levels were significantly decreased, whereas plasma FFA levels significantly reduced in the DM + ALA50, DM + ALA100, and DM + ALA200 groups, respectively, compared with those in the DM group (Table 1).

**TABLE 1:** Profiles of fasting serum glucose and insulin, plasma triglyceride, and free fatty acid levels in high‐fat diet and streptozotocin‐induced type 2 diabetic rats after treated with alpha‐lipoic acid for 13 weeks

	Normal	DM	DM + Pi	DM + ALA50	DM + ALA100	DM + ALA200
Fasting serum glucose (mg/dl)	76.4 ± 6.4^c^	274.1 ± 20.1^a^	238.0 ± 29.1^b^	266.7 ± 19.8^a^	247.1 ± 15.4^b^	252.3 ± 4.0^b^
Fasting serum insulin (μg/L)	1.10 ± 0.24^ab^	1.72 ± 0.27^a^	0.67 ± 0.16^b^	0.85 ± 0.37^ab^	0.76 ± 0.17^b^	0.72 ± 0.16^b^
Fasting plasma triglyceride (mg/dl)	89.0 ± 23.2^c^	250.7 ± 29.7^a^	103.2 ± 32.7^c^	163.3 ± 14.9^b^	184.7 ± 6.7^b^	104.0 ± 18.9^c^
Fasting plasma free fatty acid (mmol/L)	0.82 ± 0.12^ab^	1.00 ± 0.29^a^	0.72 ± 0.24^b^	0.70 ± 0.09^b^	0.76 ± 0.18^ab^	0.73 ± 0.09^b^

Note

**FIGURE 1:**
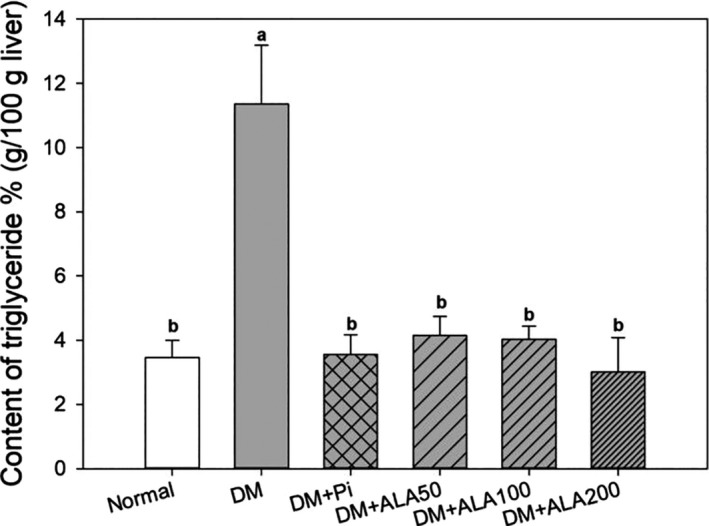
Liver triglyceride levels in high‐fat diet and streptozotocin‐induced type 2 diabetic rats treated with alpha‐lipoic acid for 13 weeks. Normal: normal diet; DM: high‐fat diet (HFD; 60% fat) plus STZ (30 mg/kg body weight, i.p.) induced diabetic rats; DM + Pio: DM rats gavaged with pioglitazone (30 mg/kg body weight) for 13 weeks; DM + ALA50: DM rats gavaged with ALA (50 mg/kg body weight) for 13 weeks; DM + ALA100: DM rats gavaged with ALA (100 mg/kg body weight) for 13 weeks; DM + ALA200: DM rats gavaged with ALA (200 mg/kg body weight) for 13 weeks. a‐b letters indicate significant differences among all samples tested (*p* < .05)

Abbreviations are as in Figure 1. a‐c letters indicate significant difference among tested samples in the same row (*p* < .05).

### 3.2 Effects of ALA on hepatic lipid levels in T2DM rats

The respective TG contents in the liver of the normal, DM + Pio, DM + ALA50, DM + ALA100, and DM + ALA200 groups were 3.45 ± 0.54, 3.56 ± 0.62, 4.14 ± 0.59, 4.02 ± 0.41, and 3.01 ± 1.07 g/100 g liver, whereas the DM group exhibited 11.35 ± 0.84 g/100 g liver (Figure 1). The liver TG contents were significantly decreased in the DM + ALA50, DM + ALA100, and DM + ALA200 groups when compared with that in the DM group.

### 3.3 Effects of ALA on NLRP3 inflammasome‐related proteins and IL‐1β protein expression in the liver of T2DM rats

**FIGURE 2:**
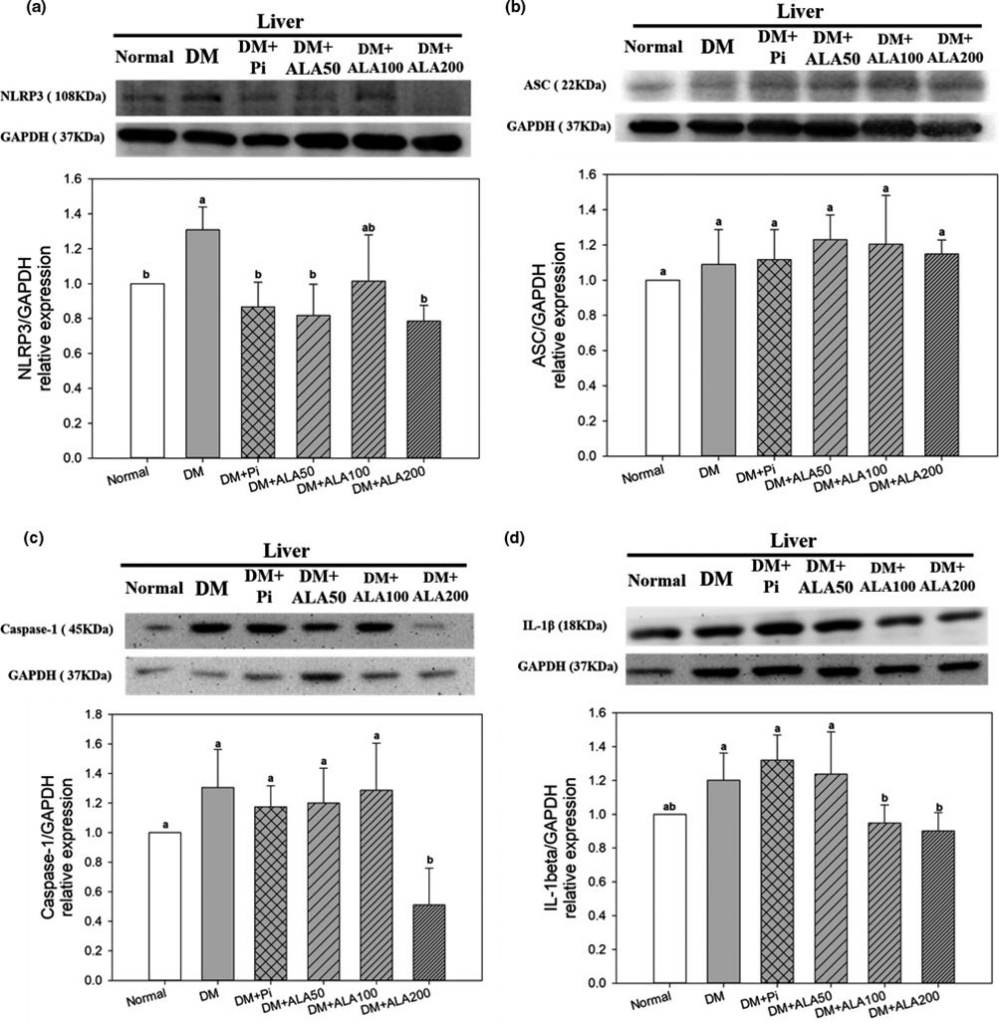
Protein expression levels of NLRP3 (a), ASC (b), caspase‐1 (c), and IL‐1β (d) in the livers of high‐fat diet and streptozotocin‐induced type 2 diabetic rats treated with alpha‐lipoic acid for 13 weeks. Abbreviations are as in Figure 1. a‐c letters indicate significant difference among all samples tested (*p* < .05)

The NLRP3 inflammasome is one of the most important inflammasomes because it affects chronic liver inflammation in humans. The effects of ALA treatment on hepatic NLRP3 inflammasome‐related proteins and IL‐1β expression in T2DM rats are shown in Figure 2. The protein expression levels of hepatic NLRP3 in DM + ALA200 groups were significantly decreased by 40.0%, respectively, compared with that in the DM group (Figure 2a). However, the treatment of ALA did not alter the hepatic ASC protein expression levels in the DM rats (Figure 2b). Additionally, the respective expression levels of hepatic caspase‐1 in DM + ALA200 groups were significantly reduced by 60.8% (Figure 2c), whereas the IL‐1β levels in DM + ALA100 and DM + ALA200 groups were significantly decreased by 21.1% and 25.0%, respectively, compared with that in the DM group (Figure 2d).

### 3.4 Effects of ALA on NF‐κB expression and ALT/AST ratio in the liver of T2DM rats

**FIGURE 3:**
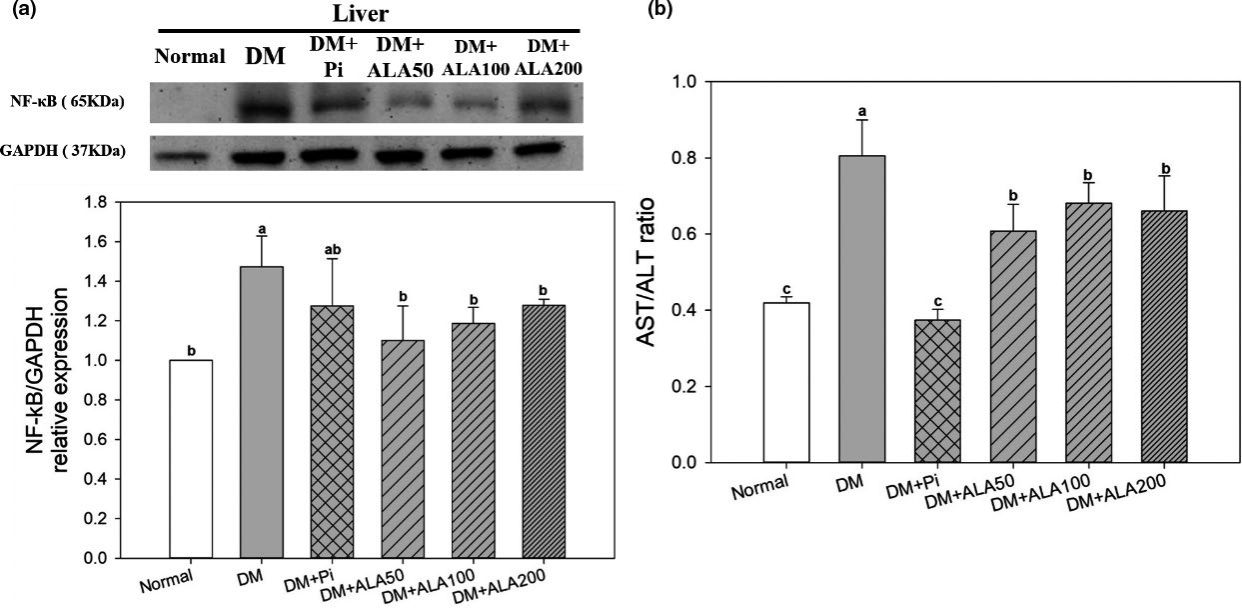
Liver protein expression levels of NF‐κB (a) and AST/ALT ratio (b) of high‐fat diet and streptozotocin‐induced type 2 diabetic rats treated with alpha‐lipoic acid for 13 weeks. Abbreviations are as in Figure 1. a‐b letters indicate significant difference among all samples tested (*p* < .05)

The protein expression levels of hepatic NF‐κB in the DM + ALA50, DM + ALA100, and DM + ALA200 groups were decreased by 25.2%, 19.4%, and 13.2%, respectively, in comparison with the DM group (Figure 3a). On the other hand, the hepatic ALT and AST values of the DM group were 81.8 ± 12.9 (U/L) and 64.3 ± 15.1 (U/L), respectively (data not shown). The treatment of ALA increased the values of hepatic ALT and AST in DM rats (data not shown). Moreover, the ALT/AST ratio in DM + ALA50, DM + ALA100, and DM + ALA200 groups was significantly decreased by 24.5%, 15.4%, and 18.0%, respectively, compared with that in the DM group (Figure 3b).

### 3.5 Effects of ALA on insulin signaling‐related protein expression in the liver of T2DM rats

**FIGURE 4:**
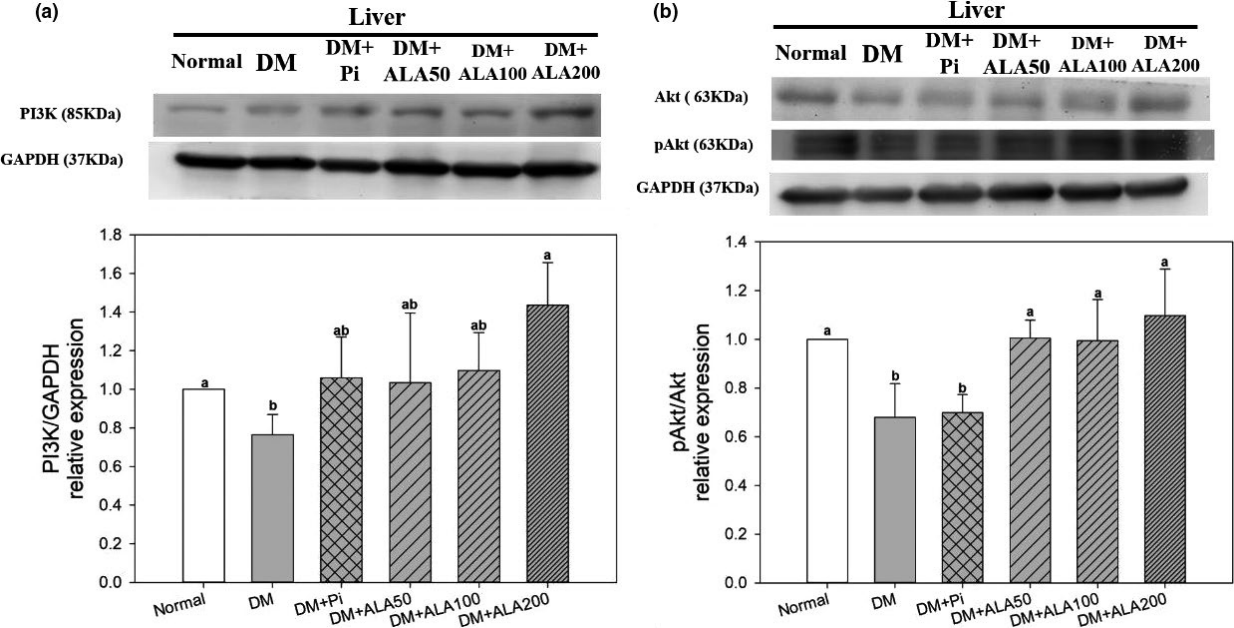
Protein expression levels of PI3K (a) and Akt (b) in the livers of high‐fat diet and streptozotocin‐induced type 2 diabetic rats treated with alpha‐lipoic acid for 13 weeks. Abbreviations are as in Figure 1. a‐b letters indicate significant difference among all samples tested (*p* < .05)

The protein expression levels of hepatic PI3K in the DM + ALA200 groups were increased by 87.7% compared with that in the DM group (Figure 4a). In addition, the protein expression level of hepatic pAkt/Akt in the DM + ALA200 groups was increased by 61.2% compared with that in the DM group (Figure 4b).

### 3.6 Effects of ALA on SREBP‐1c and CPT‐1 protein expression in the liver of T2DM rats

**FIGURE 5:**
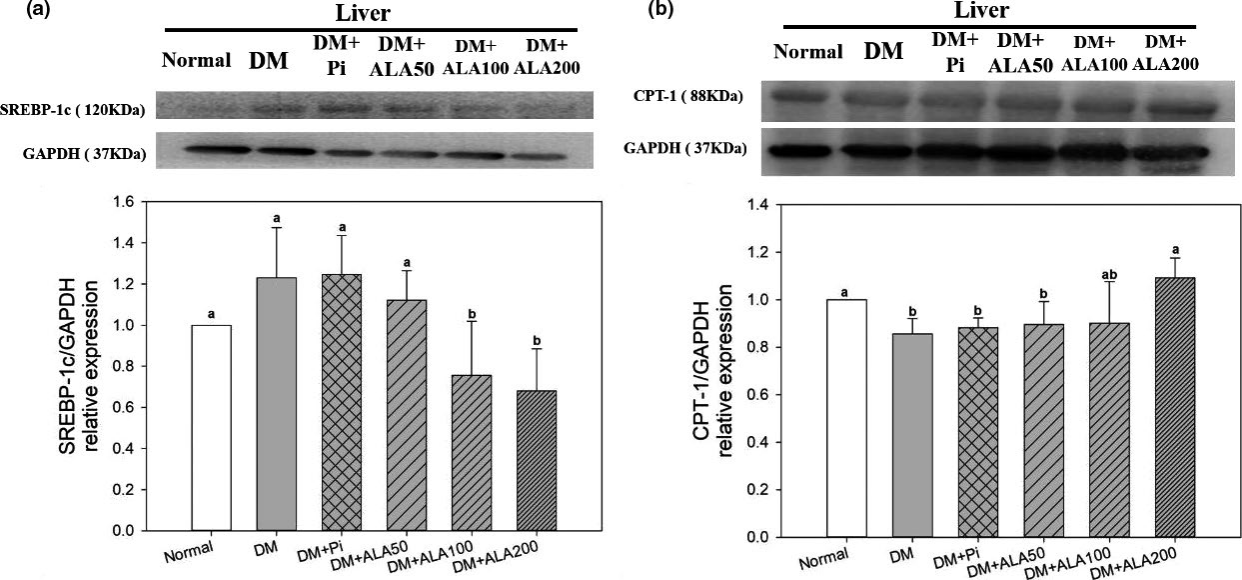
Protein expression levels of SREBP‐1c (a) and CPT‐1 (b) in the livers of high‐fat diet and streptozotocin‐induced type 2 diabetic rats treated with alpha‐lipoic acid for 13 weeks. Abbreviations are as in Figure 1. a‐b letters indicate significant difference among all samples tested (*p* < .05)

The protein expression levels of hepatic SREBP‐1c in the DM + ALA100 and DM + ALA200 groups were significantly decreased by 39.4% and 45.5%, respectively, compared with that in the DM group (Figure 5a). In addition, the protein expression level of hepatic CPT‐1 in the DM + ALA200 groups was increased by 27.5% compared with that in the DM group (Figure 5b).

## 4 DISCUSSION

Excessive calories or fat intake can cause lipid cells apoptosis, promote the activation of the inflammatory c‐Jun N‐terminal kinase 1 pathway, increase the secretion of cytokines, such as tumor necrosis factor (TNF)‐α or IL‐1β, and block insulin signaling in liver tissue, consequently resulting in compensatory insulin production by pancreatic β‐cells and the subsequent development of hyperinsulinemia (He et al., 2011). NAFLD is defined as the presence of macrovesicular steatosis that TG level exceeding 5% in liver tissue of nondrinking individuals (Huang et al., 2020; Loomba & Sanya, 2013). The results of the present study indicate that NAFLD was induced in the HFD/STZ‐treated rats. Patients with metabolic syndrome or T2DM are commonly accompanied by the high prevalence of NAFLD (Kotronen & Yki‐Järvinen, 2008), while the occurrence of hepatic IR or lipid accumulation has been associated with the progression of T2DM and NAFLD (Chalasani et al., 2012). The results of this study also suggested that ALA treatments were able to improve hyperinsulinemia, reduce hepatic TG contents, and alleviate NAFLD in an HFD plus STZ‐induced diabetic rat model.

The NLRP3 inflammasome in hepatic Kupffer cells, a specific macrophage found in liver tissue, has been reported to be associated with the progression of NAFLD (Camellet al., 2015). The activation of the NLRP3 inflammasome stimulates the production of the proinflammatory cytokine IL‐1β and causes the occurrence of IR and inflammation (Maedler et al., 2009; Schroder et al., 2010). NLRP3 inflammasome activation requires two stimulation steps: (a) Damage‐associated molecular pattern (DAMP) or pathogen‐associated molecular pattern (PAMP) molecules trigger NLRP3 protein expression, and the NLRP3 protein combines with ASC and pro‐caspase‐1 to form the NLRP3 inflammasome; and (b) the NLRP3 inflammasome is further activated by free radicals, large crystals, or DAMPs/PAMPs, to promote the formation of caspase‐1, which then converts pro‐IL‐1β into IL‐1β, subsequently resulting in the inflammatory response (Shao et al., 2015).

To date, the literature regarding the inhibitory effects of ALA on NLRP3 has been limited. In this study, treatments with ALA significantly were found to decrease the protein expression levels of NLRP3, caspase‐1, and IL‐1β in the livers of T2DM model rats. In addition, increased NF‐κB protein expression may promote the activation of the NLRP3 inflammasome in the liver. The expression level of the upstream transcription factor NF‐κB was also significantly decreased by ALA treatment in the livers of T2DM model rats. ALA treatments did not affect hepatic ASC protein expression in T2DM model rats. The combination of NLRP3 protein and ASC, a downstream binding protein of NLRP3, may cause oligomerization, attracting downstream pro‐caspase‐1 to form the NLRP3 inflammasome, further promoting inflammatory reactions. However, when NLRP3 protein expression decreased, NLRP3 protein levels were insufficient for oligomerization with free ASC, resulting in decreased downstream caspase‐1 expression and IL‐1β production. The results of the present study indicated that ALA may inhibit the expression of NF‐κB, suppressing the activation of the NLRP3 inflammasome, and subsequently reducing cytokine production and inflammatory responses in the livers of T2DM model rats.

Hepatic IR promotes TG accumulation in the liver (Brown & Goldstein, 2008). The present study suggested that ALA alleviated hepatic IR and liver‐free fatty acid contents by inhibiting the secondary activation signal of the NLRP3 inflammasome and reducing cytokine production in the livers of T2DM model rats. The PI3K/Akt pathway is a critical pathway for insulin signal transduction. Excessive HFD consumption may result in lipid accumulation in liver cells and decreased hepatic PI3K/Akt protein expression, contributing to NAFLD formation in rats (Han et al., 2010). The cytokine IL‐1β not only destroys pancreatic β‐cells, but it also inhibits PI3K/Akt expression in the insulin signaling pathway (Stienstra et al., 2010). The administration of ALA increased the expression levels of hepatic PI3K/Akt, thus reducing IR in the livers of T2DM model rats.

Accumulation of lipids in the liver may accelerate the occurrence of NAFLD. Hepatic TG contents have been associated with lipid synthesis and oxidation in the liver. SREBP‐1c is an important transcription factor associated with fat synthesis that promotes lipid synthesis by converting acetyl‐CoA to malonyl‐CoA in the nucleus (Chao et al., 2019). The overexpression of SREBP‐1c in the liver accelerates the occurrence of NAFLD in T2DM. In this study, ALA treatments blocked the PI3K/Akt pathway, inhibited SREBP‐1c expression, and, subsequently, reduced adipogenesis in the livers of T2DM model rats. CPT‐1 is the primary regulatory enzyme for fatty acid β‐oxidation. ALA treatments also promoted hepatic CPT‐1 protein expression levels, indicating accelerated lipid metabolism in the livers of T2DM model rats. In addition, the generation of free radicals is important for secondary signal activation during the progression of NAFLD. Free radicals are primarily produced by mitochondria, such that mitochondrial dysfunction promotes increased free radical formation. AST has been reported to be released when mitochondria are damaged in the liver (Botros & Sikaris, 2013). Clinically, the AST/ALT ratio has been used to assess liver inflammation. An AST/ALT ratio above 1 indicates chronic inflammation in the liver (Botros & Sikaris, 2013). In the current study, T2DM model rats exhibited the highest AST/ALT ratio, whereas ALA treatment reduced the AST/ALT ratio, indicating that ALA may reduce the secondary signaling associated with hepatic NLRP3 inflammasome activation in the livers of T2DM model rats. The above observations suggested that ALA may reduce inflammatory cytokine secretion by inhibiting hepatic NLRP3 inflammasome activation, suppressing IR and lipid accumulation in the liver, and, subsequently, alleviating the occurrence of NAFLD in T2DM.

## 5 CONCLUSIONS

**FIGURE 6:**
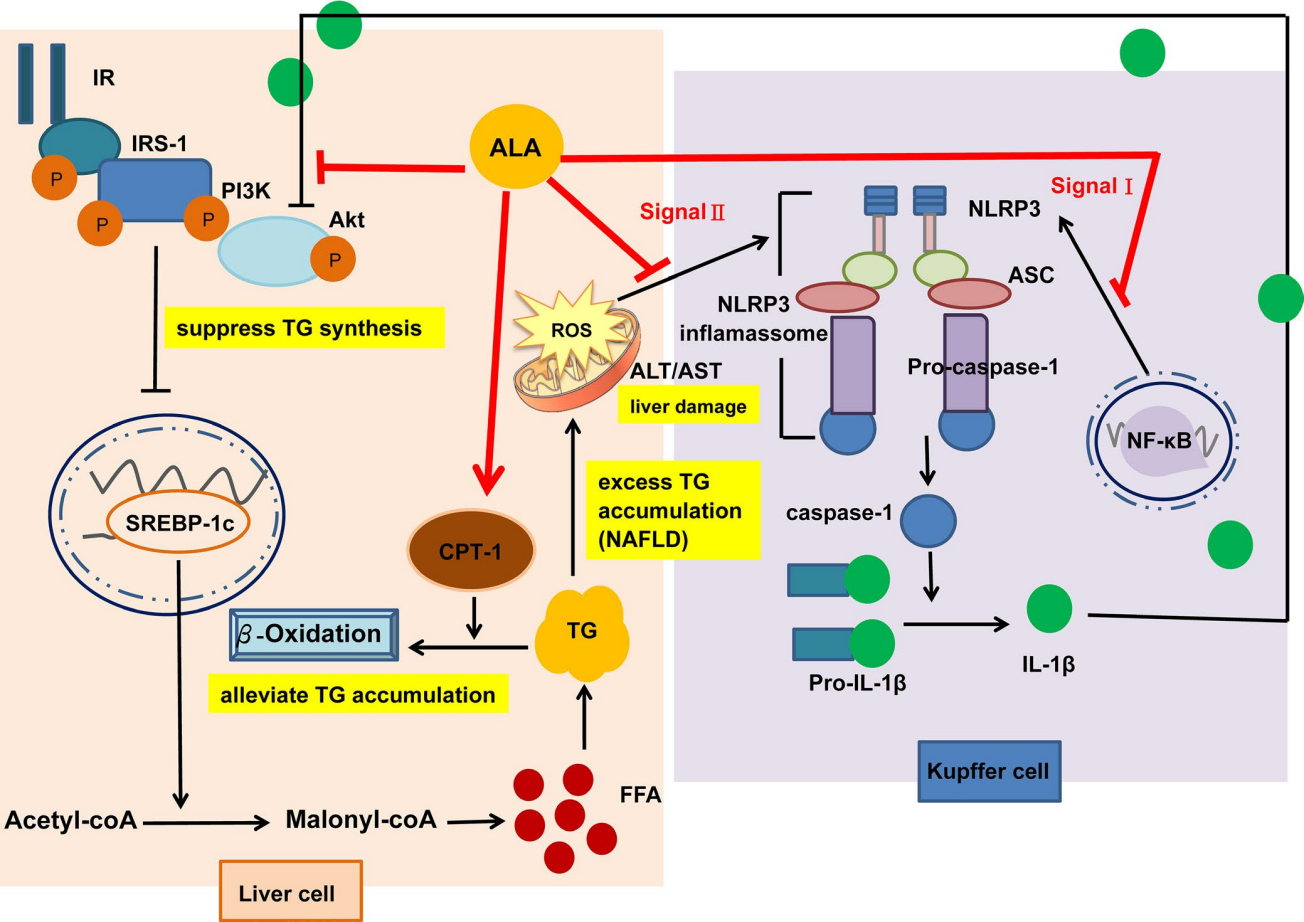
Postulated mechanism through which alpha‐lipoic acid alleviates hepatic triglyceride accumulation by suppressing the NLRP3 inflammasome pathway, suppressing lipid generation, and promoting lipid oxidation in high‐fat diet and streptozotocin‐induced type 2 diabetic rats

This study demonstrated that ALA treatment for 13 weeks was able to inhibit the activation of the hepatic NLRP3 inflammasome in T2DM rats and could be attributed to (a) decreased expression of transcription factor NF‐κB, which reduced the expression levels of NLRP3 and caspase‐1; and (b) decreased ALT/AST ratio, which reduced the production of the inflammatory cytokine IL‐1β in the liver. Moreover, ALA treatment increased PI3K/Akt protein expression levels, suppressed expression of lipid synthesis transcription factor SREBP‐1c, and increased the protein expression of lipid oxidative enzyme CPT‐1, subsequently alleviating TG accumulation in the livers of T2DM rats (Figure 6). The results of this study also suggested that ALA might be used as a health supplement for alleviation of NAFLD progression associated with T2DM.

## CONFLICT OF INTEREST

The authors have no conflict of interest to declare.

## ETHICAL APPROVAL

The study was conducted in accordance with the ethical guidelines of the Institutional Animal Care and Use Committee of National Taiwan Normal University, Taipei, Taiwan (Approval Number 106,042).

## Supporting information

Supplementary Material

## Data Availability

Research data are not shared.
