# Supplementary material for: Alpha‐lipoic acid alleviates NAFLD and triglyceride accumulation in liver via modulating hepatic NLRP3 inflammasome activation pathway in type 2 diabetic rats
Source: Food Sci Nutr. 2021 Mar 13;9(5):2733–42. doi: 10.1002/fsn3.2235 (PMC8116866; doi:10.1002/fsn3.2235)
Supplement: Supplementary file 1 — Supplementary Material [file FSN3-9-2733-s001.pdf]

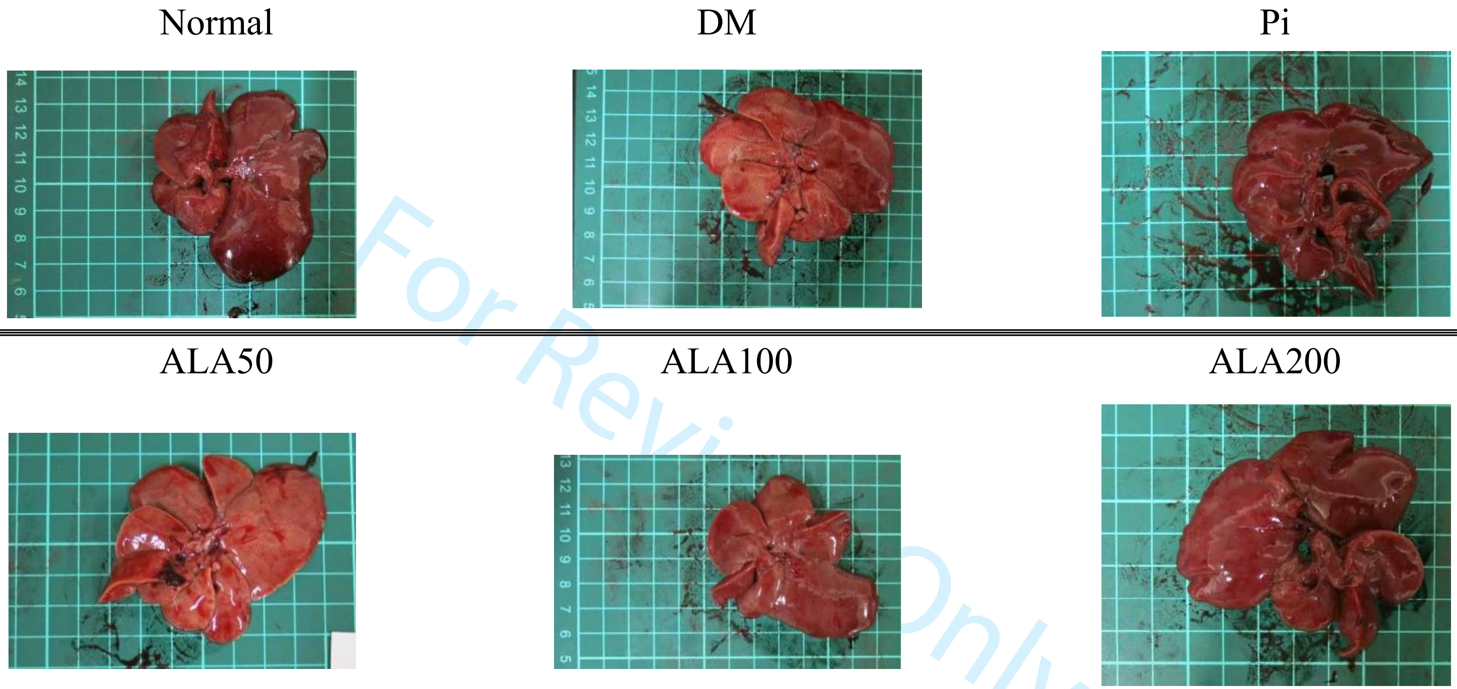

Supplementation figure 1. The exterior of liver tissue in HFD plus STZ induced T2DM rats treated with alpha-lipoic acid for 13 weeks.

For Review Only

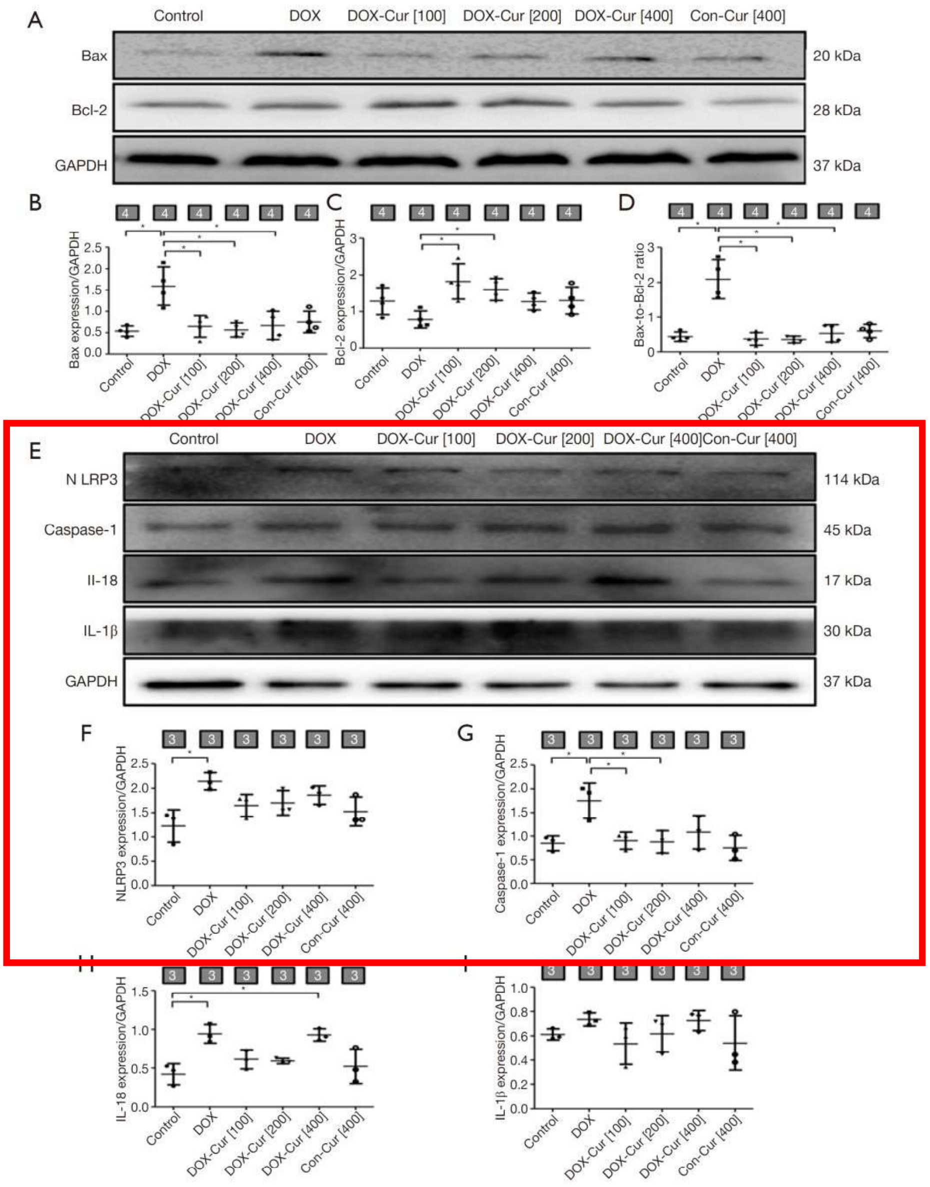

Figure 2 Effect of Cur on apoptosis and pyroptosis protein markers in DOX-treated mice. (A) Representative gel blots of Bcl-2 and Bax proteins using specific antibodies (GAPDH as loading control); (B) Bax; (C) Bcl-2; (D) Bax-to-Bcl-2 ratio; (E) representative gel blots of NLRP3, Caspase-1, IL-18 and IL-1β proteins using specific antibodies expression (GAPDH as loading control); (F) NLRP3; (G) Caspase-1; (H) IL-18; and (I) IL-1β. Mean ± SD, n=3–4 per group, *, P<0.05 between the indicated groups. Bcl-2, B-cell lymphoma-2; GAPDH, glyceraldehyde-3-phosphate dehydrogenase; Bax, Bcl-2 associated X protein; NLRP3, NLR family pyrin domain containing 3; IL-18, interleukin-18; IL-1β, interleukin-1β.

Supplementation figure 2. Effect of Cur on apoptosis and pyroptosis protein markers in DOX-treated mice.

Reference:

Yu, W., Qin, X., Zhang, Y., Qiu, P., Wang, L., Zha, W., & Ren, J. (2020). Curcumin suppresses doxorubicin-induced cardiomyocyte pyroptosis via a PI3K/Akt/mTOR-dependent manner. *Cardiovascular Diagnosis and Therapy*, *10*(4), 752-769.
